# Sonoelastographic evaluation of plantar fascia after shock wave therapy for recalcitrant plantar fasciitis: A 12-month longitudinal follow-up study

**DOI:** 10.1038/s41598-020-59464-8

**Published:** 2020-02-13

**Authors:** Chueh-Hung Wu, Yun-Yi Lin, Wen-Shiang Chen, Tyng-Guey Wang

**Affiliations:** 1 Department of Physical Medicine and Rehabilitation, National Taiwan University Hospital, College of Medicine, National Taiwan University, Taipei, Taiwan; 2 Institute of Health Policy & Management, College of Public Health, National Taiwan University, Taipei, Taiwan; 3 Department of Rehabilitation Medicine, Da-Chien Hospital, Miao-Li, Taiwan

**Keywords:** Outcomes research, Musculoskeletal abnormalities, Ligaments

## Abstract

Extracorporeal shockwave therapy (ESWT) is proposed to be effective in reducing pain and improving functional outcome in chronic plantar fasciitis. However, no long-term reports exist on the changes in plantar fascia (PF) elasticity after ESWT. We aimed to evaluate the changes in PF stiffness in patients with plantar fasciitis undergoing ESWT. The visual analogue scale (VAS, 0–100) was used for evaluating heel pain severity. B-mode sonography and strain sonoelastography were used for evaluating the PF thickness and stiffness. The sonoelastogram was analyzed using hue histogram analysis (value: 0–255, from stiffer to softer). All evaluations were recorded before ESWT, and 1 week, 1 month, 3 months, 6 months, and 12 months after ESWT. Repeated measures ANOVA was used to compare pain VAS, PF thickness, and PF hue value at different follow-up time-points. Twenty-two participants (8 men, 14 women) completed all measurements for 12 months. The VAS of heel pain, PF thickness, and PF hue values at pre-ESWT, and 1-week, 1-month, 3-month, 6-month, and 12-month evaluations after ESWT were 62.4 ± 4.2, 49.3 ± 5.8, 38.3 ± 5.7, 27.9 ± 5.3, 18.9 ± 4.7, and 13.2 ± 3.0 (*p* < 0.01 in all measurements post ESWT versus pre-ESWT); 5.57 ± 0.22 mm, 5.64 ± 0.18 mm, 5.45 ± 0.24 mm, 5.37 ± 0.20 mm, 5.08 ± 0.20 mm, and 4.62 ± 0.15 mm (*p* < 0.01 at 6-month; otherwise *p* > 0.05); and 24.5 ± 2.4, 35.2 ± 3.1, 31.0 ± 4.1, 30.5 ± 3.9, 21.4 ± 2.1, and 15.9 ± 1.6 (*p* < 0.01 at 1-week and 6-month; otherwise *p* > 0.05), respectively. In conclusion, the heel pain intensity and PF thickness reduced gradually over 12 months after ESWT. The PF stiffness decreased during the first week and increased thereafter; at the 12-month follow-up, stiffness was more than at pre-ESWT.

## Introduction

Plantar fasciitis is the most common cause of inferior heel pain in adults^1^. If a patient’s heel pain lasts over 6 months despite conservative treatment, it is considered chronic recalcitrant plantar fasciitis^2^. In such patients, low-intensity extracorporeal shockwave therapy (ESWT) has been proposed to be effective in reducing pain and improving functional outcome^3^. While the exact mechanism of ESWT remains unknown, animal experiments have demonstrated that ESWT induces neovascularization at the tendon-bone junction, which might imply improvement of blood supply and tissue regeneration^4,5^. Through this regeneration process, there might be certain material property changes of the plantar fascia (PF), but such changes have not been described.

Morphologically, a significant decrease in PF thickness after ESWT was revealed by using B-mode sonography^6,7^. In addition to morphology changes, regeneration of the PF may be also associated with changes of its elasticity. However, there has been no report on long-term follow-up of PF elasticity changes after ESWT. To achieve such evaluation, an ultrasound-based technique, strain sonoelastography provides an estimation of soft tissue elasticity. Previous studies using this technology have shown softening of the PF in plantar fasciitis patients^8–13^. Therefore, in this prospective assessor-blind longitudinal follow-up study, we aimed to evaluate elasticity changes of the PF after ESWT by using strain sonoelastography. We hypothesized that softened PF would become stiffer after ESWT.

## Materials and Methods

### Participants

This prospective longitudinal follow-up study was approved by the Research Ethics Committee, National Taiwan University Hospital (Ref. No.: 201007055R). Each participant provided written informed consent before evaluation, and the work was completed in accordance with the principles laid down by the *Declaration of Helsinki*. The participants were recruited between September 7, 2010 and January 28, 2014.

Inclusion criteria of the participants were (1) age 20–80 years old; (2) unilateral heel pain at the insertion of the PF on the medial tubercle of the calcaneus; (3) worse pain when waking up in the morning or after rest; (4) pain duration longer than 6 months despite conservative treatments including shoe modification, arch support, medication and physiotherapy; (5) visual analogue scale (VAS) of worst heel pain in previous one week >40 on a scale of 100; and (6) B-mode ultrasound (US) examination revealing a thickened (>4 mm) and hypoechoic PF. Exclusion criteria were (1) having received steroid or hyaluronic acid injections to the heel; (2) having undergone shock wave therapy or operations on the heel; (3) a history of systemic inflammatory diseases.

### Extracorporeal shock wave therapy

Piezoson100 (Richard Wolf, Knittlingen, Germany), a piezoelectric-type device, was used by one physiatrist for ESWT. All participants received three sessions of ESWT (3000 shock waves per session of 0.08–0.2 mJ/mm^2^) at weekly intervals. No local anesthesia was applied during treatment. The target of treatment was determined by the self-reported tender area. A sonographic examination was performed only before each session of ESWT to confirm the depth of the PF. The shock waves were applied to the maximum pain sites and to the surrounding area within a 1-cm radius.

### Ultrasound and sonoelastographic examination

The examination of each PF included B-mode US scanning and strain sonoelastography using an Acuson S2000 US system (Siemens, Munich, Germany) with a 6–14-MHz linear transducer (14L5; Siemens). US imaging settings (transducer frequency: 11 MHz, total gain: +20 dB, focus: 1.5 cm in depth, dynamic range: 70, SieClear (SC): 2, map: B, space/time (ST): 0) were set to standardize each measurement. All examinations were conducted by one physiatrist, with a 6-year experience of musculoskeletal ultrasound.

Each participant was examined in a prone position with 90° of knee flexion in the neutral ankle position^8,14^. The ultrasound transducer can be hold more steadily in this posture, which is important for obtain high-quality strain sonoelastography images. The entire width of the PF was examined to localize the thickest area. In a longitudinal view, the PF thickness was measured from the anterior edge of the inferior calcaneal border vertically to the inferior border of the PF. Sonoelastograms of the PF were obtained three times in the same position with a quality factor exceeding 60, indicating stable transducer positioning.

The stiffness color scale used in the sonoelastogram expresses differing degrees of tissue stiffness with corresponding colors^15^. For the Siemens system, the scale indicates the relative stiffness of the examined tissues within the region of interest (ROI) and ranges from red (hardest), yellow (relatively hard), green (intermediate stiffness), blue (relatively soft), to purple (softest).

### Hue histogram analysis of sonoelastogram

**Figure 1:**
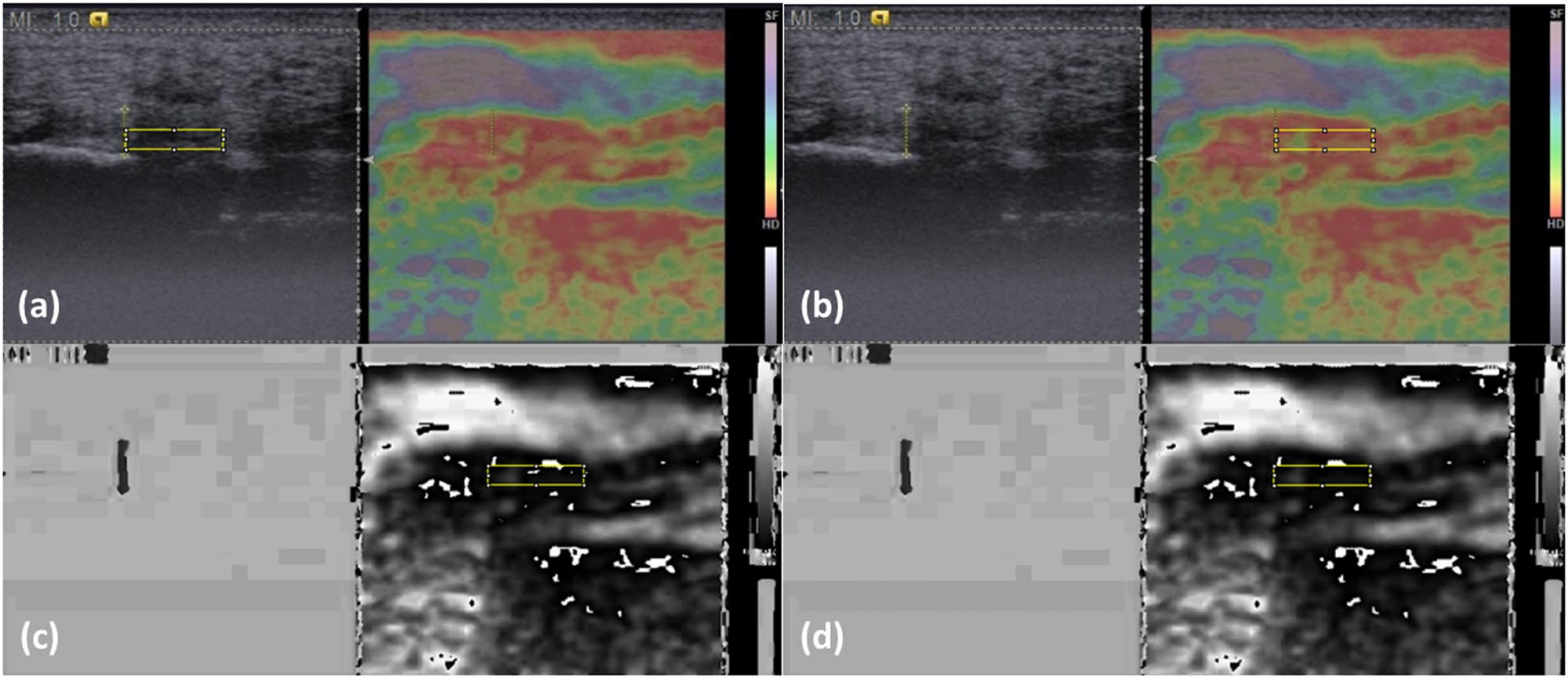
Image processing for RGB color histogram and hue histogram analyses. (**a**) Select a region of interest (ROI) in the B-mode image, (**b**) move the ROI to the same place on the sonoelastogram for color histogram processing, (**c**) convert the color-coded sonoelastogram into hue component of the HSB system, and (**d**) following noise signal correction, obtain the hue histogram of all pixels within the ROI.

Image J software (version 1.43 u; National Institutes of Health, Bethesda, USA) was used to acquire an RGB color histogram and a hue histogram of a manually-selected standardized rectangular ROI for each PF sonoelastogram. Since the site of abnormality in plantar fasciitis mostly occurs near the PF origin at the medial tuberosity of the calcaneus^16^, the longer side of the ROI was set 1 cm distal to the proximal PF insertion on the distal calcaneus border, and the shorter side was set 2 mm toward the heel from the point of the inferior calcaneus border. This was done to avoid including the area outside the PF since most normal PFs are thicker than 2 mm^17^ (see Fig. 1a, the yellow rectangular delineated area).

In RGB color histogram analysis, we used the Image J “Color Histogram” function to analyze all pixels within the ROI. The red, green, and blue color intensities (ranging from 0 to 255) of each pixel were calculated separately and averaged as mean values. Such analysis may be superior to dichotomizing the PF stiffness into hard and soft solely based on the percentage of blue area^18^.

In hue histogram analysis, sonoelastograms were converted from the original RGB coded images (Fig. 1b) to the hue component of hue, saturation, and brightness (HSB) coded images using the “HSB Stack” function. The originally-colored sonoelastograms and stiffness color scale were thus converted to monotonic gray scale images (Fig. 1c) in which each hue value (0–255) represented a different level of relative stiffness within the ROI, with 0 representing the hardest and 255 representing the softest. Following conversion, images contained some black or white noise signals at the softest and hardest parts (Fig. 1c). These noise signals resulted from violations of the monotonically increasing rule during conversion from RGB to HSB color-coding systems according to the conversion equations^19^. We corrected these noise signals by assigning those pixels to hue value of 0 in the hardest region and value 255 in the softest region. The post-processing image is shown in Fig. 1d. We then used the “Histogram” function to obtain the average hue value of all pixels within the ROI to represent the average relative stiffness. A higher hue value indicated a relatively lower stiffness. The results of the hue histogram and RGB color histogram analyses of the three images from the same patient were averaged for statistical analysis.

### Outcome measurements and follow-up

VAS of heel pain, PF thickness, and PF elasticity (hue value) were recorded before ESWT, and 1 week, 1 month, 3 months, 6 months, and 12 months after ESWT.

### Statistical analysis

MedCalc Statistical Software version 19.0.5 (MedCalc Software bvba, Ostend, Belgium; https://www.medcalc.org; 2019) was used for all statistical analysis. Because a preliminary Kolmogorov–Smirnov test demonstrated that all samples followed a normal distribution, repeated measures ANOVA was used to compare pain VAS, PF thickness, and PF hue value among different timings of follow-ups. A *p* value of <0.05 was considered as a statistically significant difference. All data were expressed as mean ± standard error of the mean.

## Results

Thirty-one subjects were recruited for assessment of eligibility. Twenty-nine received ESWT treatment (two were excluded due to not meeting inclusion criteria). One was lost to follow-up at 3 months, three at 6 months, and three at 12 months. Twenty-two participants (8 men, 14 women; age 52.3 ± 1.6 years; height 163.6 ± 2.2 cm; weight 71.1 ± 4.1 kg; body mass index 26.2 ± 1.0 kg/m^2^; symptom duration: 18.6 ± 4.2 months) completed all measurements for 12 months. All participants tolerated ESWT intensity, and no focal ecchymosis or other significant adverse effects were observed during follow-ups.

**Figure 2:**
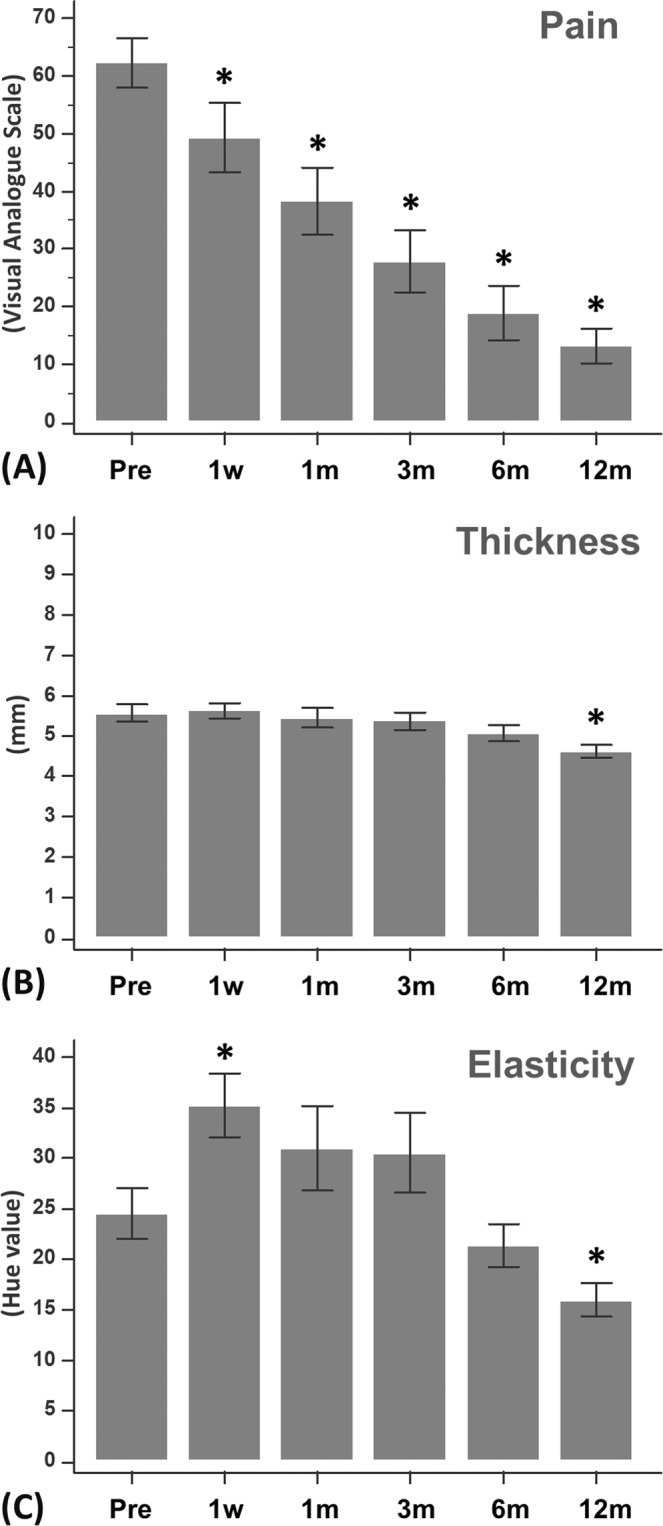
Outcome measurements before and after extracorporeal shock wave therapy (ESWT). (Pre: before ESWT; 1w: 1 week after ESWT; 1 m: 1 month after ESWT; 3 m: 3 months after ESWT; 6 m: 6 months after ESWT; 12 m: 12 months after ESWT) (*p < 0.01 versus before ESWT). (**A**) Visual analogue scale score for heel pain (0–100). (**B**) Plantar fascia thickness (mm). (**C**) Plantar fascia elasticity (hue value).

The results of VAS of heel pain, PF thickness, and PF hue values before ESWT, and at 1 week, 1 month, 3 months, 6 months, and 12 months after ESWT were shown in Table 1. According to these values, the PF became softer one week after ESWT and had similar stiffness to pre-ESWT at 1-month, 3-month, and 6-month follow-ups. The PF became stiffer than pre-ESWT 12 months after ESWT. (Fig. 2).

**Table 1:** Heel pain and ultrasonographic evaluations before and after ESWT.

	Before ESWT	1 week	1 month	3 months	6 months	12 months
VAS (0–100)	62.4 ± 4.2	49.3 ± 5.8*	38.3 ± 5.7*	27.9 ± 5.3*	18.9 ± 4.7*	13.2 ± 3.0*
PF thickness (mm)	5.57 ± 0.22	5.64 ± 0.18	5.45 ± 0.24	5.37 ± 0.20	5.08 ± 0.20	4.62 ± 0.15*
PF hue values	24.5 ± 2.4	35.2 ± 3.1*	31.0 ± 4.1	30.5 ± 3.9	21.4 ± 2.1	15.9 ± 1.6*

*p < 0.01 versus before ESWT (repeated measures ANOVA).

ESWT: extracorporeal shock wave therapy; VAS: visual analogue scale of heel pain; PF: plantar fascia.

Note: a higher hue value indicates a softer plantar fascia.

## Discussion

After ESWT for plantar fasciitis, heel pain intensity decreased gradually, while the PF thickness became thinner at the 12-month follow-up. Contrary to our hypothesis that the PF would become stiffer gradually after ESWT, it became softer initially at the 1-week follow-up but became stiffer than pre-ESWT at the 12-month follow-up. In an MR imaging study, increased soft-tissue and perifascial edema was observed in 75% of participants 24 hours after ESWT, while the signal intensity and PF thickness were minimally affected by ESWT^20^. In a histopathological study on the tendons of ponies, the amount of irregularly distributed tenocytes, loss of the regular collagen wave pattern, loss of the organization of fibrils, and percentage of degraded collagen were significantly increased 3 hours after ESWT^21^. There are no previous reports on the longitudinal follow-up of PF elasticity changes after three sessions of ESWT. In the current study, we found that the PF became softer one week after ESWT, which may be related to edematous changes and disorganization of the tenocytes and collagen wave pattern^20,21^. This may indicate a structurally weaker PF in the first few weeks after ESWT treatment.

Although the PF became softer at 1 week after ESWT, its stiffness returned to pre-ESWT status at 1-month, 3-month, and 6-month intervals, and it became significantly stiffer after 12 months of ESWT. The PF is a fibrous tissue composed primarily of type I collagen, similar to tendons. In a study on rats with the collagenase-induced Achilles tendinitis, biomechanical and biochemical characteristics of healing tendons were restored 12 weeks after ESWT^22^. Increasing TGF-beta 1 expression in the early stage of tendon repair and elevated IGF-I expression persisting throughout the healing period were also observed^22^. In another rat study, the associated tenocyte proliferation was reported to be mediated by early up-regulation of PCNA and TGF-beta 1 gene expression, endogenous NO release, synthesis of TGF-beta 1 protein, and collagen synthesis (at the 7th day)^23^. The above findings may indicate that ESWT metabolically “activated” these tenocytes and significantly induced them to proliferate^24^. Unlike animals, our study showed that it may take a longer time for the human PF to regain stiffness and even become stiffer.

The initially softer PF after ESWT may have important clinical implications. In the current study, we found that heel pain intensity decreased gradually, which was consistent with the effectiveness of ESWT on plantar fasciitis reported previously^25^. Despite the decrease in symptoms in the first few weeks after ESWT, the PF may be structurally weaker in this period. If the patients return to previous activity level, exerting too much pressure on the PF without adequate protection, the symptoms may quickly recur. Although we did not evaluate the effectiveness of an in-sole, we still recommend that after ESWT the patients should be instructed to wear an in-sole and to avoid excessive pressure on the heel for at least for one month, based on the results that the PF may be softer in this period. Such a protection strategy may prevent recurrence of plantar fasciitis.

US evaluation of morphological changes of PF after ESWT has been reported^6,26^. An ultrasonographic study of plantar fasciitis at 6 months follow-up after extracorporeal shock wave therapy showed that the PF thickness decreased from 5.2 ± 1.5 mm to 4.4 ± 1.0 mm at 24 weeks of follow-up^6^. In the current study, there was no statistically significant difference in PF thickness between pre-ESWT and 6 months post-ESWT, despite a trend towards a thinner PF at the 6-month follow-up. However, at the 12-month follow-up the decrease in thickness of the PF was significant. The longer time for the PF to become thinner in this study may be related to the thicker PF before ESWT, and/or a longer symptom duration (18.6 months in our study; not mentioned in the ultrasonographic study^6^).

Based on the above findings, we believe that ESWT is effective in treating chronic recalcitrant plantar fasciitis and may modify the PF morphology, with the effect lasting for 12 months. In addition to morphological changes, our study further showed elasticity changes of PF after ESWT. A sonoelastographic study revealed hardening of the PF 3 months after collagen injection for plantar fasciitis^27^. In this longitudinal follow-up study, we found an initially softer PF which became stiffer at one-year follow-up, despite a gradually decreased heel pain intensity.

Several limitations need to be addressed. First, this was a single-center, unblinded study with no control group, making it difficult to clarify whether elasticity changes were associated with natural recovery. However, in this study we included those patients with symptoms for at least 6 months despite conservative treatment. It is unlikely that the thickness and elasticity remained unchanged for 18.6 months (the mean symptom duration in this study) and then naturally recovered in the following 12 months. Second, unlike a manually marked ROI, a rectangular ROI for elastogram analysis in this study may not fit the PF well because it was smaller than the PF. Such ROI may not represent the entire PF. However, we actually conducted comparisons within the same subject at different timings. This standardized ROI may minimize bias due to hand-draw errors. Third, we did not perform pathohistological correlation in this study. Surgery is rarely necessary in plantar fasciitis, so it is difficult to obtain PF specimens. Because in previous studies a softer PF was observed in plantar fasciitis patients. In this study, the change of elasticity after ESWT was our major aim for investigation. Fourth, we utilized strain sonoelastography with image analysis, which was a semi-quantitative evaluation of tissue elasticity. Shear wave ultrasound elastography (SWUE) can provide objective evaluation of tissue elasticity by providing quantification of shear wave velocity within the tissue^28,29^. Investigation of PF stiffness with SWUE should be conducted in future studies. Because evaluation with SWUE requires the certain ultrasonography system with a particular probe, we thought that few clinicians or researchers had access to such machines. This study may provide an alternative for follow-up of tissue elasticity with utilizing a more easily accessible system.

## Conclusion

After ESWT for plantar fasciitis, heel pain intensity decreased gradually, while the PF thickness became thinner at the 12-month follow-up. The PF became softer at 1 week of follow-up and regained stiffness thereafter, finally becoming stiffer than pre-ESWT at 12 months of follow-up.
